# Chronic urticaria and quality of life

**DOI:** 10.6026/973206300221705

**Published:** 2026-03-31

**Authors:** Rabia Alam, K Harsha Vardhan, Shitij Goel, Alok Jindal

**Affiliations:** 1 Department of Dermatology, School of Medical Sciences and Research Sharda University, Greater Noida, Uttar Pradesh, India

**Keywords:** Chronic urticaria (CU), quality of life, severity, spontaneous urticaria, Urticaria Activity Score

## Abstract

Chronic urticaria (CU), particularly chronic spontaneous urticaria (CSU), is a persistent dermatological condition that significantly impacts patients' physical, emotional and social well-being. Therefore, it is of interest to assess the severity of symptoms in CU patients and evaluate their effects on daily life activities and quality of life. A cross-sectional design was employed to collect data from 109 participants, examining sociodemographic, clinical and psychosocial factors. Thus, we show that CU's severity strongly correlates with impairments in work, sleep and social relationships. This research advances knowledge by integrating clinical severity with detailed patient-reported outcomes, providing a comprehensive understanding of CU's broader impact on daily functioning.

## Background:

Chronic urticaria (CU) is a dermatological condition characterized by the recurrent appearance of transient wheals, pruritus and sometimes angioedema, persisting for six weeks or more [1]. It is primarily classified into chronic spontaneous urticaria (CSU), where no external trigger can be identified and chronic inducible urticaria (CIndU), which is triggered by specific physical or environmental factors. Although CU is not life-threatening, its unpredictable nature and persistent symptoms often lead to significant functional and psychological distress [2]. The global burden of CU is substantial, affecting up to 1% of the population at any given time, with a higher incidence observed in women, particularly those in the third to fifth decades of life. Similar demographic trends have been reported across different geographical settings, including Portugal, Vietnam, Brazil and India, where females consistently constitute the majority of the affected population [3]. CU typically presents with pruritic wheals and in approximately 30-50% of cases, with angioedema as a co-manifestation. These symptoms can be spontaneous or exacerbated by triggers such as heat, pressure or stress, though spontaneous urticaria remains the predominant form. The recurrent and relapsing nature of the condition contributes to patient frustration and complicates its management, particularly in refractory cases where symptoms persist despite standard treatment protocols [4]. The concept of quality of life (QoL) has gained significant importance in recent years, especially among medical professionals. It can be characterized as a person's contentment or enjoyment with life in areas that they deem significant, such as employment, housing and financial worries [5]. One of these aspects is health, leading to the creation of the term "health-related quality of life" (HRQoL), which describes how a patient feels their illness, affects their capacity to live life fully. HRQoL is a subjective assessment of how a patient perceives their health situation and its impact on their overall well-being [6]. In recent decades, the incorporation of patients' perceptions into disease management has become essential for improving QoL in healthcare models. Traditional clinical parameters often fail to capture the subjective experiences of patients, leading to increased interest in HRQoL within the scientific community [7]. CU significantly interferes with subjective well-being and daily life. Some patients with CU report health status comparable to individuals with coronary artery disease and severe asthma. CU also causes significant disruption in family structures and affects work, school and leisure activities [8]. Skin pruritus in CU can lead to discomfort such as sleep disturbances, exhaustion, drowsiness and insomnia, commonly caused by the condition itself or the side effects of antihistamines. Psychiatric diseases like depression, hysteria, hypochondria and post-traumatic stress disorder are also highly prevalent among CU patients [9]. Additionally, patients with CU often complain of recurrent pain syndromes such as tension headaches and fibromyalgia. The degree to which QoL is affected by CU varies according to the disease's etiology and severity. When CU is associated with delayed pressure urticaria, it typically has a more significant impact on QoL than other forms of urticarial [10]. Therefore, it is of interest to evaluate the correlation between the clinical severity of chronic urticaria and the quality of life of patients.

## Methodology:

This study was a cross-sectional and analytical design conducted over 18 months, from April 2024 to November 2025. The study was carried out in the Department of Dermatology, Venereology and Leprosy at Sharda Hospital, Greater Noida. Ethical clearance was obtained from the ethics committee before the initiation of the study. All patients presenting with symptoms of urticaria lasting more than 6 weeks and clinically diagnosed with chronic urticaria, were included in the study.

## Sample size calculation:

The sample size was calculated using the following formula:

N=Z2.P.(1-P)D2N = \frac{Z^2 \cdot P \cdot (1 - P)}{D^2}N=D2Z2.P.(1-P) 

## Where:

Z=1.96Z = 1.96 Z=1.96 (Standard normal variate),

P=16.65% P = 16.65\% P=16.65% (Prevalence),

D=7% D = 7\% D=7% (Precision).

By applying these values, the sample size calculated was 109 patients, which were included in the study. Patient selection included adult and adolescent patients with symptoms of urticaria lasting more than 6 weeks and those who were willing to participate in the study. Pregnant women, children, and immunocompromised patients were excluded. After obtaining ethical approval, 109 patients diagnosed with chronic urticaria who met the study criteria were enrolled. The following information was collected from each participant: informed consent, demographic details such as name, age, sex, address, marital status, and socioeconomic status, a detailed clinical history including the onset and evolution of the disease, a general physical and systemic examination, and a detailed cutaneous examination, including body surface area calculation. Patients were then asked to fill out the Dermatology Life Quality Index (DLQI) questionnaire. The questionnaire was available in both English and Hindi and in cases where the patient could not read or write, an interview was conducted verbally and the appropriate responses were recorded.

## Statistical analysis:

The data collected was compiled in Microsoft Excel and analyzed using SPSS Version 26. The distribution of quantitative data was assessed using the Kolmogorov-Smirnov test. Central tendency and dispersion were expressed using mean and standard deviation for quantitative variables, while categorical variables were expressed in terms of frequency and percentages, along with graphical representations. The association between categorical variables was analyzed using the Pearson Chi-square test. A p-value of 0.05 or less was considered statistically significant. This methodology was designed to ensure robust and reliable data collection and analysis, supporting the study's objective of understanding chronic urticaria and its impact on patients' quality of life.

## Results:

The sociodemographic characteristics of the study participants (N = 109) has been shown in this table, in terms of age distribution, the largest group consisted of participants aged 36-45 years, comprising 35 individuals (30.8%), followed by 31 participants (29.0%) aged 26-35 years. Both the 15-25 and 46-55 age groups included 16 participants each (15.0%), while 11 participants (10.3%) were over the age of 55. Regarding educational attainment, the majority of participants were educated (n=80, 81.7%) whereas the rest were illiterate (n=20, 18.3%) Occupationally, the largest proportions of respondents were employed in private organizations, accounting for 38 participants (33.6%). This was followed by 30 self-employed individuals (28.0%), 23 working in government organizations (21.5%), 12 students (11.2%) and 6 unemployed individuals (5.6%). A substantial majority of the participants, 94 (86.0%), reported living in urban areas, while 15 (14.0%) resided in rural locations. Socioeconomic classification using the Kuppuswamy scale showed that 57 participants (51.4%) belonged to the lower-middle class, while 38 (35.5%) were from the upper-middle class. The remaining respondents included 5 participants each (4.7%) from the upper-lower and upper classes and 4 participants (3.7%) from the lower class. With respect to family size, 50 participants (44.9%) belonged to families comprising 4-7 members. Additionally, 39 participants (36.4%) reported having more than seven members in the family, while 20 (18.7%) came from families with 1-4 members. Lastly, marital status data revealed that 70 participants (63.6%) were married, whereas 39 (36.4%) were unmarried (Table 1 - see PDF). The descriptive statistics for key continuous variables in the study is shown in this table the mean age of participants was 38.79 years with a standard deviation of 12.35, indicating a moderate spread of ages across the sample. The average number of members in a participant's family was 6.85 (SD = 2.64), reflecting relatively large household sizes in the sample population. The mean age of onset of the condition under investigation was 37.59 years, with a standard deviation of 12.68, suggesting that onset occurred primarily in mid-adulthood but with considerable variability (Table 2 - see PDF).

The clinical characteristics related to the duration, onset and distribution of skin lesions among the study participants (N = 109) has been shown in the table, the duration of complaints was most commonly reported as either up to 6 months or between 6 months and 1 year, with 41 participants (38.3%) falling into each category. A smaller number of participants reported a duration of 1 to 2 years (10 participants; 7.5%) or more than 2 years (17 participants; 15.9%). The age of onset varied across the sample, with the highest number of participants 37 individuals (32.7%) reporting onset between 26 and 35 years of age. This was followed by 30 participants (28.0%) whose symptoms began between 36 and 45 years. Eighteen participants (16.8%) experienced onset between the ages of 15 and 25 years, 14 (13.1%) between 46 and 55 years and 10 (9.3%) reported onset after the age of 55. Regarding the distribution of lesions, nearly all participants (108 individuals; 99.1%) presented with generalized lesions, while only 1 participant (0.9%) had localized lesions. The site of initial onset varied, with the chest, abdomen or back being the most frequently reported (33 participants; 29.0%), followed by the whole body (31 participants; 28.8%). Other reported sites included the arms (22 participants; 20.6%), hands (8 participants; 7.5%), face (7 participants; 6.5%), legs (4 participants; 3.7%), neck (2 participants; 1.9%) and feet (1 participant; 0.9%) (Table 3 - see PDF). The medical history and comorbid conditions reported by the participants (N = 109) has been shown in the table, the majority of participants (105 individuals; 96.3%) described their condition as progressive, while only 4 participants (3.7%) reported a stable condition. A family history of similar complaints was noted in 6 participants (5.6%). Regarding comorbidities, hyperthyroidism was the most frequently reported, affecting 22 participants (20.6%). Diabetes mellitus was present in 9 individuals (8.4%), whereas hypertension was reported by only 1 participant (0.9%), while the rest did not have any comorbidity. These findings indicate that while most participants experienced a progressive course of illness, comorbid endocrine conditions, particularly hyperthyroidism, were relatively common within the sample (Table 4 - see PDF). The Urticaria Activity Score (UAS) measured over a span of days showed a mean value of 35.13, accompanied by a standard deviation of 8.76, indicating a moderate level of disease activity among the participants (Table 5 - see PDF). The mean Urticaria Activity Score (UAS) over days according to the severity of complaints among participants has been shown in the table, those with symptoms lasting between 6 months and 1 year (n = 41) had a mean UAS of 34.35 with a standard deviation of 9.14. Similarly, those with complaints of up to 6 months' duration (n = 41) exhibited a comparable mean UAS of 33.95 (SD = 9.45), indicating a similar level of disease activity in the early stages. Notably, higher mean UAS scores were observed among participants with a longer duration of illness. Those with symptoms persisting for 1 to 2 years (n = 10) had a mean score of 38.50 (SD = 5.86), while participants with complaints extending beyond 2 years (n = 17) also recorded a mean UAS of 38.50 (SD = 6.00). These findings suggest that longer disease duration may be associated with higher urticaria activity levels (Table 6 - see PDF).

The severity of symptoms reported by participants (N = 109) in relation to itching, wheals and swelling of the eyes and lips in them has been shown in the table, with regard to itching, the majority of participants 74 individuals (69.2%) reported being "very much" troubled by the symptom. This was followed by 24 participants (22.4%) who reported being troubled "a lot," 7 (4.7%) who said "rather," and 4 (3.7%) who were only "a little" affected. Similarly, wheals were reported as "very much" troublesome by 66 participants (61.7%), while 31 (28.0%) reported being affected "a lot." A smaller number of participants felt troubled "rather" (7 individuals; 4.9%) or "a little" (5 individuals; 4.5%). Then asked about swelling of the eyes, 77 participants (72.0%) reported not experiencing this symptom at all. However, 24 participants (22.4%) indicated they were "a little" troubled, followed by 5 (2.8%) who said "rather," 2 (1.9%) who were affected "a lot," and 1 participant (0.9%) who reported being "very much" troubled. Swelling of the lips was also not experienced by most participants, with 81 individuals (74.8%) stating "not at all." Nevertheless, 21 participants (19.6%) reported being "a little" affected, while smaller numbers reported being affected "rather" (4 participants; 2.1%), "a lot" (2 participants; 1.7%) or "very much" (1 participant; 0.9%). These findings indicate that itching and wheals were the most severe and commonly experienced symptoms, while swelling of the eyes and lips were reported by fewer participants and generally with less severity. The relationship between the severity of itching symptoms and the Urticaria Activity Score (UAS) measured over days among the participants (N = 109) has been shown in the table, who reported being "very much" troubled by itching (n = 74) had the highest mean UAS score of 38.59 with a standard deviation of 6.48, indicating a strong association between intense pruritus and elevated disease activity. Those who reported being troubled "a lot" (n = 24) had a lower mean UAS of 28.29 (SD = 8.63), while participants who responded with "rather" (n = 7) recorded a mean score of 23.80 (SD = 3.83). Interestingly, the "a little" category (n = 4) had a slightly higher mean UAS of 26.25 (SD = 10.50) compared to the "rather" group, though this small sample size may limit interpretation. These findings suggest a positive association between the severity of itching and UAS scores, with higher pruritus intensity reflecting greater disease activity in chronic urticaria patients. The association between the severity of wheals experienced by participants and their corresponding Urticaria Activity Scores (UAS) over days has been shown in the table, among those who reported being "very much" troubled by wheals (n = 66), the mean UAS was 38.82 with a standard deviation of 6.04, indicating the highest disease activity within this group. Participants who reported being troubled "a lot" (n = 30) had a lower mean UAS of 30.33 (SD = 9.43), while those in the "rather" category (n = 7) recorded a mean score of 28.00 (SD = 8.57). The lowest mean UAS was observed in participants who were only "a little" troubled by wheals (n = 5), with a mean score of 25.20 (SD = 9.39). This trend reinforces a clear gradient: as the subjective severity of wheals increases, so does the objective measure of disease activity, underscoring the clinical relevance of patient-reported symptom burden in chronic urticarial. The relationship between the severity of swelling of the lips is discussed along urticaria Activity Scores (UAS) over days among study participants, the majority of participants (n = 81) who did not report any lip swelling had a mean UAS of 34.04 with a standard deviation of 9.10. Among those who were "a little" troubled by swelling (n = 21), the mean UAS was slightly higher at 37.33 (SD = 7.45), suggesting a modest increase in disease activity. Participants who reported being troubled "a lot" (n = 2) or "rather" (n = 4) by swelling had a mean UAS of 42.00 in both cases, with no variability (SD = 0), indicating consistently high symptom severity within these small groups. The single participant who reported being "very much" affected also had a UAS of 42.00; however, the standard deviation was not applicable due to the sample size of one. These findings suggest that the presence and severity of lip swelling are associated with higher UAS scores, although the small sample sizes in the more severe categories warrant cautious interpretation. The extent to which symptoms impacted various aspects of daily life and activities among participants (N = 109) has been shown in this Table 7 (see PDF), among this a substantial portion of participants (57 individuals; 53.3%) reported that their work life was "very often" affected by their symptoms, while others experienced limitations "often" (21 participants; 19.6%), "sometimes" (17; 15.9%) or "rarely" (14; 11.2%). Physical activities were also notably impacted, with 49 participants (44.9%) stating they were affected "very often," 26 (24.3%) "Often," 10 (7.5%) "Sometimes" and 24 (22.4%) "Rarely". This highlights a significant burden on functional mobility and exercise. Sleep disturbances were common, with over half of the participants (55; 50.5%) reporting that their symptoms "often" interfered with sleep. Additionally, 28 (26.2%) experienced sleep problems "rarely," 13 (12.1%) "sometimes," 7 (6.5%) "very often," and only 6 (3.7%) reported "never" being affected. Social relationships were impacted to varying degrees, with 38 participants (35.5%) reporting "often" being affected, followed by 32 (29.9%) who experienced it "rarely," 22 (20.6%) "sometimes," 4 (1.9%) "very often," and 13 (12.1%) not affected at all. Finally, in terms of eating habits, 34 participants (31.8%) reported being "sometimes" affected, 32 (29.9%) "rarely," 21 (19.6%) "often," and 3 (0.9%) "very often." However, 19 individuals (17.8%) indicated their eating habits were "never" impacted by symptoms. These findings collectively underscore that chronic urticaria symptoms significantly disrupt daily functioning, particularly in domains of work, physical activity and sleep.

The association between the extent to which participants' work life was affected by their symptoms and their corresponding Urticaria Activity Scores (UAS) over days is depicted in this table, amongst which participants who reported being "very often" affected in their work (n = 57) had the highest mean UAS score of 40.16 with a standard deviation of 4.29, indicating a strong correlation between symptom severity and work impairment. Those who were "often" affected at work (n = 21) had a slightly lower mean UAS of 37.00 (SD = 6.69), while participants who reported being affected only "sometimes" (n = 17) or "rarely" (n = 14) exhibited much lower mean UAS scores of 23.06 (SD = 4.12) and 25.08 (SD = 8.15), respectively. The association between limitations in social relationships and Urticaria Activity Scores (UAS) over days among participants is discussed here, amongst the patients those who reported being "very often" affected (n = 2) had the highest possible mean UAS score of 42.00 with no variation (SD = 0), indicating consistently severe disease activity. A similarly high mean UAS was observed among participants who were "often" affected (n = 38), with a score of 40.71 (SD = 3.20). Participants who reported "sometimes" being affected in their social relationships (n = 22) also had elevated scores, with a mean UAS of 38.82 (SD = 5.17). In contrast, those who were "rarely" affected (n = 32) had a significantly lower mean UAS of 29.09 (SD = 9.43), while participants who experienced no social limitations (n = 13) had the lowest mean score of 26.38 (SD = 7.64). These results suggest a strong positive correlation between urticaria severity and social impairment, emphasizing the psychosocial impact of the condition. Impact of symptoms on food choices and Urticaria Activity Scores (UAS) over days among participants. The highest mean UAS was recorded in the "very often" category (n = 3) with a score of 42.00, though no standard deviation could be calculated due to the single observation. Participants who reported being affected "often" (n = 21) had a nearly identical mean UAS of 41.00 (SD = 2.51), reflecting high disease activity levels. Those affected "sometimes" (n = 34) had a mean score of 39.94 (SD = 4.40), also indicating significant impact. In contrast, participants who were affected "rarely" (n = 32) had a considerably lower mean UAS of 30.84 (SD = 9.72), while those who reported "never" being affected in their eating habits (n = 19) had the lowest mean score of 26.89 (SD = 7.48). The reported difficulties and quality of life issues experienced by participants (N = 109) as a result of urticaria is discussed in this table. A substantial proportion of participants reported sleep-related disturbances. Over half (57 participants; 53.3%) reported having "very often" experienced difficulty falling asleep, with an additional 21 (19.6%) experiencing it "sometimes." Similarly, 46 participants (43.0%) reported waking up at night "very often," while another 23 (21.5%) each reported it occurring "often" or "rarely." Difficulties with concentration were frequently reported as well; 52 participants (48.6%) reported this "often," and another 10 (9.3%) "very often." Only 6 participants (3.7%) stated they "never" had difficulty concentrating. Dietary restrictions due to urticaria were reported by many, with 42 participants (39.3%) stating that they "sometimes" had to limit their food choices and 25 (23.4%) experiencing it "rarely." Public embarrassment was another psychosocial impact; 50 participants (46.7%) reported feeling embarrassed to go to public places "often," while 30 (28.0%) experienced this "rarely" and 4 (3.7%) "very often." Overall, these findings reveal that chronic urticaria significantly impacts various dimensions of daily life ranging from sleep and emotional health to diet, social functioning and physical activity with a notable proportion of participants reporting moderate to severe interference. The association between reported difficulty falling asleep and Urticaria Activity Scores (UAS) over days among study participants is depicted in this table, where the highest mean UAS was observed among those who reported having this difficulty "very often" (n = 57), with a mean score of 40.89 and a standard deviation of 2.90. Participants who experienced difficulty "often" (n = 17) had a slightly lower but still elevated mean UAS of 39.67 (SD = 4.32). In contrast, significantly lower UAS scores were reported by participants who experienced this difficulty "sometimes" (n = 21), with a mean of 24.67 (SD = 5.25) and those who experienced it "rarely" (n = 14), with a mean UAS of 22.50 (SD = 2.98). These findings highlight a strong positive relationship between increasing sleep initiation difficulties and greater disease severity, as measured by UAS. Here the relationship between the frequency of nocturnal awakenings and Urticaria Activity Scores (UAS) over days among participants is depicted here, those who reported waking up at night "often" (n = 23) had the highest mean UAS score of 41.09 (SD = 2.41), closely followed by those who experienced it "very often" (n = 46), with a mean score of 40.78 (SD = 3.06). In contrast, markedly lower UAS scores were recorded among those who reported waking up "rarely" (n = 23), with a mean score of 22.52 (SD = 3.63) and those who experienced it "sometimes" (n = 14), with a mean of 28.50 (SD = 6.42). The single participant who reported "never" waking up at night had a UAS of 21 (SD = 0.1). These results suggest a strong association between nocturnal disturbances and higher urticaria activity, emphasizing the considerable impact of sleep fragmentation on disease severity. Here we have discussed the relationship between self-reported difficulty concentrating and Urticaria Activity Scores (UAS) among participants, The highest mean UAS score was reported by participants who experienced concentration problems "very often" (n = 10), with a score of 42.00 and no variability (SD = 0). Those who experienced this symptom "often" (n = 52) also had an elevated mean UAS of 41.06 (SD = 2.78), suggesting a strong link between cognitive interference and urticaria severity. Participants who experienced concentration issues "sometimes" (n = 13) had a moderate mean UAS of 34.46 (SD = 6.04), while those who reported "rarely" experiencing it (n = 28) had a mean of 23.75 (SD = 5.16). The lowest UAS score was recorded among those who "never" had concentration difficulties (n = 6), with a mean of 22.75 (SD = 3.50). These findings indicate a strong positive correlation between cognitive symptoms and disease severity, further reinforcing the multidimensional burden of chronic urticaria on patients' daily functioning. The association between feelings of embarrassment related to urticaria and Urticaria Activity Scores (UAS) over days is discussed, in which the Participants who reported being "very often" embarrassed to go to public places (n = 4) had a maximum UAS of 42.00 with no variability (SD = 0). Those who felt "often" embarrassed (n = 50) also reported a high mean UAS of 41.02 (SD = 2.45), indicating severe disease activity closely tied to social self-consciousness. Participants who reported being embarrassed "sometimes" (n = 16) had a slightly lower mean UAS of 38.50 (SD = 6.76), whereas those who were "rarely" embarrassed (n = 30) had a considerably lower mean UAS of 25.43 (SD = 6.23). The lowest UAS scores were reported among those who were "never" embarrassed (n = 9), with a mean of 23.00 (SD = 3.42). These findings reflect a strong positive association between social embarrassment and urticaria severity, emphasizing the psychosocial toll the condition can have on individuals' public and interpersonal engagement.

## Discussion:

This study on chronic urticaria (CU), particularly chronic spontaneous urticaria (CSU), provides valuable insights into the severity of symptoms and their impact on quality of life, aligning with findings from previous research. Dias *et al.* (2016) [10] similarly highlighted the significant impairment of quality of life in CU patients, especially in domains like sleep, emotional health and daily activities, with higher disease severity correlating to worse quality of life. The present study also supports this, emphasizing the multidimensional burden of CU, which includes not only physical symptoms but also psychosocial factors such as emotional distress, sleep disruption and social limitations. Maurer *et al.* (2020) [11] found that CU often remains active despite treatment, a trend observed in this study as well, where patients reported persistent symptoms and poor disease control, as measured by UAS7. Additionally, Sharma *et al.* (2025) [12] corroborated the findings of this study, as both studies observed a significant relationship between symptom severity and poorer quality of life, particularly for those with angioedema. Hollis *et al.* (2018) [13] confirmed the reliability and consistency of the UAS7 scoring system, which was used effectively in this study to stratify disease severity. Lastly, Keller *et al.* (2024) [14] discussed the importance of combining clinical measures with patient-reported outcomes, a methodology that was integral to this study, which linked clinical severity to psychosocial impacts. Together, these studies reinforce the notion that CU is not only a physical ailment but also a condition with profound emotional and social consequences, supporting the need for integrated care approaches that address both the clinical and psychosocial aspects of the disease.

## Conclusion:

Chronic urticaria (CU) significantly impacts patients' physical, emotional and social well-being, with symptom severity directly correlating to quality of life impairments. Thus, we show the importance of integrated care that addresses both clinical management and psychosocial support. Future research should continue exploring the multifaceted burden of CU and refine treatment strategies for better patient outcomes.
